# In this Issue

**Published:** 2012

## Overview: Assessing the Genetic Risk for Alcohol Use Disorders

Genetic factors determine a substantial portion of a person’s risk for alcoholism. Drs. Tatiana Foroud and Tamara J. Phillips summarize some of the approaches that have been used to determine the magnitude of the overall genetic contribution to alcohol dependence in specific populations and identify particular genes involved. Traditional approaches in humans include linkage analyses, case–control studies, and genome-wide association studies. Approaches using animal models of human alcoholism have focused on targeted breeding strategies and the generation of animals in which specific genes are deleted or inactivated. Most recently, investigators are using approaches analyzing the genetic basis of alcoholism at the level of the entire genome, thus moving beyond analyses of the roles of individual genes in the development alcoholism. (pp 266–272)

## Identifying Genetic Variation for Alcohol Dependence

Researchers use a wide range of analyses to identify the genes involved in the development of alcoholism and their specific roles in this process. In this article, Drs. Arpana Agrawal and Laura J. Bierut discuss the advantages and limitations of these approaches, explore the role of such genetic studies of alcoholism in the context of other coexisting diseases (e.g., esophageal cancer), and suggest additional steps such as the development of large-scale research consortia, that can help enhance the success of genetic studies and, ultimately, gain new insight into potential treatment approaches. (pp 274–282)

## Using Genetically Engineered Animal Models in the Postgenomic Era to Understand Gene Function in Alcoholism

After identifying numerous genetic variations that underlie the complex phenotype of alcoholism, researchers now need to determine how these variations translate into altered biological function. Much of this work involves genetically engineering animal models in which individual genes are deleted or inactivated, if possible, restricting gene alterations to certain tissues or developmental periods, report Drs. Matthew T. Reilly, R. Adron Harris, and Antonio Noronha. Together with high throughput genetic engineering and genome sequencing strategies that draw at least in part on community-wide resources, these strategies hopefully will lead to additional breakthroughs in understanding the genetic basis of alcoholism. (pp 282–291)

## Epigenetics—Beyond the Genome in Alcoholism

Processes that modify gene expression without altering the underlying DNA sequence (i.e., epigenetic processes) contribute to a person’s predisposition to alcoholism, report Mr. Bela G. Starkman and Drs. Amul J. Sakharkar and Subhash C. Pandey. For example, these processes modify the proteins with which the DNA binds in the cell, thereby making the DNA more or less accessible to the enzymes that are involved in gene expression. Methylation of the DNA also can interfere with gene expression. The authors describe several examples of how these and other epigenetic mechanisms influence the expression of genes related to alcoholism. (pp 293–305)

## Identifying Gene Networks Underlying the Neurobiology of Ethanol and Alcoholism

Although many DNA regions and genes have been identified that may be associated with alcoholism, researchers have not been able to place these genes in any kind of biological context that would explain the underlying functional biology. According to Drs. Aaron R. Wolen and Michael F. Miles, the emerging field of systems biology, which allows for analyses of entire gene networks, may help researchers elucidate the genetic basis of complex traits, such as alcoholism, both in humans and in animal models. The use of high-throughput technologies for molecular profiling enables researchers to identify novel gene–gene interactions and describe gene networks that may shed new light on the processes involved in the development of alcoholism. (pp 306–317)

## The Impact of Gene–Environment Interaction on Alcohol Use Disorders

There are three different gene–environment interactions—the additive model, the “fan-shaped” interaction, and the crossover interaction. In this article, Drs. Danielle M. Dick, and Kenneth S. Kendler discuss what is known about gene–environment interactions in the field of alcohol use disorders and the challenges in interpreting these three types of interactions. (pp 318–324)

## Bridging Animal and Human Models: Translating From (and to) Animal Genetics

Studying both humans and animal models is necessary to fully understand the neurobiology of alcoholism from the molecular to the cognitive level, including issues such as alcohol withdrawal severity, sensitivity to rewards, impulsivity, and dysregulated alcohol consumption. In this article, Ms. Amanda M. Barkley-Levenson and Dr. John C. Crabbe discuss how the use of animal models, such as rodents, nonhuman primates, and even invertebrates, allows for a degree of genetic and environmental control that would not be possible in human studies. By using these species to recapitulate discrete aspects of alcohol use disorders as they appear in human populations, researchers are able to target the specific biological underpinnings of the disease. (pp 325–335)

## Genes Contributing to the Development of Alcoholism: An Overview

A wide range of experimental approaches have been used to identify genes contributing to the development of alcohol dependence. In this article, Dr. Howard J. Edenberg reviews some of these strategies as well as some of the genes that have been implicated in alcoholism risk based on findings from these studies. These include genes encoding enzymes involved in alcohol metabolism, the receptor for the brain signaling molecule (i.e., neurotransmitter) γ-aminobutyric acid (GABA), proteins involved in the circadian rhythm, and proteins involved in immune responses, all of which will be explored in more detail in subsequent articles in this issue. (pp 336–338)

## Genes Encoding Enzymes Involved in Ethanol Metabolism

The genes that encode the main enzymes involved in ethanol metabolism, alcohol dehydrogenase (ADH) and aldehyde dehydrogenase (ALDH), influence a person’s risk of alcoholism. Both enzymes are encoded by several genes, some of which exist in different variants that influence the rate of ethanol and acetaldehyde metabolism. Drs. Thomas D. Hurley and Howard J. Edenberg describe specific variants in both ADH- and ALDH-encoding genes that alter ethanol metabolism in a way which impacts the drinker’s risk of alcoholism as well as of associated conditions, such as esophageal cancer. (pp 339–344)

## Alcohol Dependence and Genes Encoding α2 and γ1 GABA_A_ Receptor Subunits: Insights from Humans and Mice

One group of genes that have been implicated in the risk for alcoholism encompasses genes that encode receptors for the signaling molecule (i.e., neurotransmitter) γ-aminobutyric acid (GABA). According to Drs. Cecilia M. Borghese and R. Adron Harris, considerable evidence points to the GABAA receptor as one of the main targets of alcohol; and DNA variations (i.e., polymorphisms) in the genes encoding this receptor have been linked with alcohol dependence. The authors posit that analysis of the specific gene variants for the GABA_A_ receptor may be a first step in matching alcohol-dependent patients with appropriate pharmacotherapy. (pp 345–353)

## Immune Function Genes, Genetics, and the Neurobiology of Addiction

The immune system and those parts of the nervous system that regulate immune responses play a role in the development of addictions, particularly in the context of stressful situations. Both stress and alcohol exposure can activate certain cells of the nervous system, resulting in the induction of genes involved in innate immune responses, particularly inflammatory reactions. According to Dr. Fulton T. Crews, one pivotal component of this process is a regulatory protein called NF-κB, which is regulated by both stress and alcohol. Alcohol-related induction of innate immune genes in certain brain regions can contribute to alcohol’s effects by disrupting the decision-making processes and inducing negative emotions as well as impact alcohol drinking behavior. (pp 355–361)

## Circadian Genes, the Stress Axis, and Alcoholism

A bidirectional relationship exists between the body’s internal system controlling the body’s daily rhythm (i.e., the circadian system), with the circadian system influencing alcohol use patterns and alcohol consumption altering circadian functions. Several “clock genes” contribute to the circadian system, whose activities are tightly controlled. In this article, Dr. Dipak K. Sarkar explores why the circadian system is vulnerable to alcohol toxicity and describes the complex interactions between the circadian system, the stress response, and alcohol consumption. For example, alcohol-mediated modulation of clock genes may help modulate the activity of the body’s stress response system, which in turn may increase the propensity to drink alcohol following a stressful event. (pp 362–366)

## Discovering Genes Involved in Alcohol Dependence and Other Alcohol Responses: Role of Animal Models

Many genes play a role in the development of alcohol dependence. However, these genes do not explain all the genetic variance associated with alcoholism, and systematic approaches to gene discovery are critical to identify novel genes and mechanisms involved in alcohol dependence. Drs. Kari J. Buck, Lauren C. Milner, Deaunne L. Denmark, Seth G.N. Grant, and Laura B. Kozell describe efforts using animal models for identifying specific alcohol-related traits and that have resulted in the identification of DNA regions, quantitative trait genes (QTGs), and high-quality QTG candidates as well as their plausible mechanisms of action. These animal-derived DNA loci and QTGs may be relevant to alcoholism risk in humans. (pp 367–374)

